# Preventing *Staphylococcus aureus* Sepsis through the Inhibition of Its Agglutination in Blood

**DOI:** 10.1371/journal.ppat.1002307

**Published:** 2011-10-20

**Authors:** Molly McAdow, Hwan Keun Kim, Andrea C. DeDent, Antoni P. A. Hendrickx, Olaf Schneewind, Dominique M. Missiakas

**Affiliations:** Department of Microbiology, University of Chicago, Chicago, Illinois, United States of America; University of California, San Francisco, United States of America

## Abstract

*Staphylococcus aureus* infection is a frequent cause of sepsis in humans, a disease associated with high mortality and without specific intervention. When suspended in human or animal plasma, staphylococci are known to agglutinate, however the bacterial factors responsible for agglutination and their possible contribution to disease pathogenesis have not yet been revealed. Using a mouse model for *S. aureus* sepsis, we report here that staphylococcal agglutination in blood was associated with a lethal outcome of this disease. Three secreted products of staphylococci - coagulase (Coa), von Willebrand factor binding protein (vWbp) and clumping factor (ClfA) – were required for agglutination. Coa and vWbp activate prothrombin to cleave fibrinogen, whereas ClfA allowed staphylococci to associate with the resulting fibrin cables. All three virulence genes promoted the formation of thromboembolic lesions in heart tissues. *S. aureus* agglutination could be disrupted and the lethal outcome of sepsis could be prevented by combining dabigatran-etexilate treatment, which blocked Coa and vWbp activity, with antibodies specific for ClfA. Together these results suggest that the combined administration of direct thrombin inhibitors and ClfA-antibodies that block *S. aureus* agglutination with fibrin may be useful for the prevention of staphylococcal sepsis in humans.

## Author Summary

*Staphylococcus aureus* secretes factors that perturb blood coagulation in infected hosts. We report here that three bacterial products – coagulase (Coa), von Willebrand factor binding protein (vWbp) and clumping factor (ClfA) - act together and promote agglutination, the association of staphylococci with polymerized fibrin cables. Staphylococcal agglutination was associated with thromboembolic lesions in heart tissues and a lethal outcome of *S. aureus* sepsis in mice. Inhibition of Coa and vWbp with direct thrombin inhibitors, drugs already approved for the prevention of stroke, as well as passive transfer of antibodies specific for Coa, vWbp and ClfA could prevent the pathogenesis of *S. aureus* sepsis. These results suggest new preventive and/or therapeutic strategies that may improve the outcome of *S. aureus* sepsis in humans, a disease that is otherwise associated with high mortality.

## Introduction

The Gram-positive bacterium *Staphylococcus aureus* is the causative agent of human skin and soft tissue infections, invasive disease and bacteremia [1]. Staphylococcal bacteremia leads to endocarditis and sepsis, diseases that, even under antibiotic therapy, are associated with high mortality [2]. Community- and hospital-acquired infections are frequently caused by antibiotic (methicillin)-resistant *S. aureus* (MRSA) [3], resulting in poor disease outcomes following the failure of antibiotic therapy [4]. A preventive strategy that can reduce the burden and improve the outcomes of *S. aureus* sepsis is therefore urgently needed [5].

*S. aureus* is a unique disease pathogen owing to its multiple interactions with fibrinogen [6], [7], [8], a highly abundant host protein responsible for the formation of fibrin clots following cleavage by thrombin [9]. Fibrinogen is a glycoprotein with *M*r ∼340,000, formed by three pairs of A*α*-, B*β*-, and *γ*-chains covalently linked to form a “dimer of trimers,” where A and B designate the fibrinopeptides released by thrombin cleavage [10]. The elongated molecule folds into three separate domains, a central domain E that contains the N-termini of all six chains and two flanking domains D formed mainly by the C-termini of the B*β*- and *γ*-chains [9]. These globular domains are connected by long triple-helical structures [9].

*S. aureus* secretes two coagulases, Coa and von-Willebrand factor binding protein (vWbp), polypeptides that also promote cleavage of the Aα and Bß chains of fibrinogen to generate fibrin clots [11]. Coagulases conformationally activate the central coagulation zymogen prothrombin [10]. The crystal structure of the active complex revealed binding of the D1 and D2 domains of coagulases to prothrombin and insertion of their Ile^1^-Val^2^ N-terminus into the Ile^16^ pocket of the zymogen, inducing a functional active site through conformational change [11]. Exosite I of *α*-thrombin, the fibrinogen recognition site, and proexosite I on prothrombin are blocked by the D2 of Coa [11]. Nevertheless, association of the tetrameric (Coa·prothrombin)_2_ complex enables fibrinogen binding at a new site with high affinity [10]. This model explains the coagulant properties and efficient fibrinogen conversion by coagulases [10]. *S. aureus* mutants lacking both coagulases, *coa* and *vwb*, are unable to form abscesses in a mouse model of staphylococcal diseases [12]. When used as a purified antigen, Coa and vWbp elicit protective immune responses that prevent the formation of abscesses in the same model [12].

The coagulation of calcium-chelated plasma following incubation with bacteria [12] is still used in clinical laboratories to distinguish *S. aureus* isolates from non-pathogenic staphylococci (coagulase test) [13]. Another diagnostic tool, the slide agglutination test, monitors the agglutination of *S. aureus* immersed in calcium-chelated plasma [14]. The biochemical attributes and physiological relevance of staphylococcal agglutination are not yet known. *S. aureus* strains express clumping factor A (ClfA) [15], a surface protein that promotes precipitation of staphylococci through association with soluble fibrinogen (clumping reaction) [16], [17], [18]. The N2 and N3 domains of ClfA (residues 229–545) bind to the C-terminal end of the fibrinogen γ-chains (residues 395–411) [19], [20]. *S. aureus* mutants lacking functional *clfA* display virulence defects in mouse models for septic arthritis or endocarditis, phenotypes that have been attributed to the loss of staphylococcal binding to fibrinogen deposited on inflamed joint tissues or on mechanically damaged heart valves [21], [22]. ClfA also contributes to staphylococcal escape from phagocytic killing, which involves its binding to complement regulatory factor I [23]. A ClfA-specific monoclonal antibody has been isolated that blocks staphylococcal association with the fibrinogen γ-chain [24]. A phase II clinical trial with bacteremic patients compared the efficacy of monoclonal antibody (Tefibazumab) and antibiotic treatment with placebo and antibiotic. However, composite clinical end point analysis did not detect differences between placebo and antibody [25].

Birch-Hirschfeld employed a biochemical approach to elucidate *S. aureus* agglutination in citrate-plasma and proposed a reaction pathway involving both fibrinogen and prothrombin [26]. This work suggests a considerably more complex mechanism for *S. aureus* agglutination rather than the direct association of bacteria with fibrinogen (clumping). To explore this possibility, we have searched for staphylococcal mutants that are defective for agglutination and/or sepsis with the purpose of identifying new preventive strategies for this disease.

## Results

### Surface proteins contribute to staphylococcal sepsis

We previously developed an animal model to examine the genetic requirements for staphylococcal sepsis [27]. Briefly, *S. aureus* Newman, 1×10^8^ CFU, is injected into the retro-orbital plexus of BALB/c mice, resulting in 100% lethality over a ten day observation period [27]. This model was used to examine the contribution of secreted coagulases to staphylococcal sepsis [12]. *S. aureus* Newman mutants lacking the *coa* and *vwb* genes displayed increased time-to-death and increased survival phenotypes [12](Table 1). Earlier work identified sortase A (SrtA), an enzyme that links surface proteins to the staphylococcal cell wall envelope [28], as an essential virulence factor for sepsis [27]. Nevertheless, these studies left unresolved which surface protein(s) play a key role in this disease process. *S. aureus* mutants with insertional lesions in any one of eighteen surface protein genes [29] were tested for their role in sepsis (Table 1). These experiments identified clumping factor A (ClfA) as the single most important contributor (Table 1). Although mutations in *clfA* diminished the severity of clinical disease and improved the outcome of sepsis, *clfA* mutants retained significant virulence and were still capable of killing infected animals, unlike *srtA* variants (Table 1).

**Table 1:** Surface protein genes and their contribution to *S. aureus* sepsis.

Genotype	P values	Median survival time (hours ± SEM)
wild-type	-	24 (1.6)
*srtA*	<0.0001	>240
*sasF*	1.000	24 (1.6)
*sdrC*	0.5416	24 (1.2)
*sdrD*	0.5416	24 (1.2)
*sasD*	0.3415	24 (2.0)
*isdA*	0.3116	24 (1.8)
*sasG*	0.1462	24 (0)
*clfB*	0.0888	24 (1.2)
*sdrE*	0.0888	24 (4.8)
*isdH*	0.0143	24 (2.0)
*isdB*	0.0243	30 (3.2)
*sasA*	0.0004	36 (7.3)
*isdC*	<0.0001	36 (1.2)
*vwb*	<0.0001	36 (2.6)
*fnbpA*	0.0004	48 (5.5)
*sasB*	<0.0001	48 (7.4)
*sasC*	0.0011	54 (8.8)
*fnbpB*	<0.0001	60 (8.0)
*coa*	<0.0001	72 (12.5)
*adsA*	<0.0001	96 (16.7)
*clfA*	<0.0001	120 (15.3)

BALB/c mice were infected by retro-orbital injection with 1×10^8^ CFU of *S. aureus* Newman or its variants with insertional lesions in either sortase A (*srtA*) or any one of eighteen genes encoding sortase A-anchored surface proteins or the two coagulase genes, *coa* and *vwb*. Median survival time represents the time at which 50% of infected mice (n = 10) exhibited lethal disease. Statistical significance was determined by the two-tailed Logrank test. Data are representative of two independent experiments.

### Genetic requirements for staphylococcal agglutination

**Figure 1:**
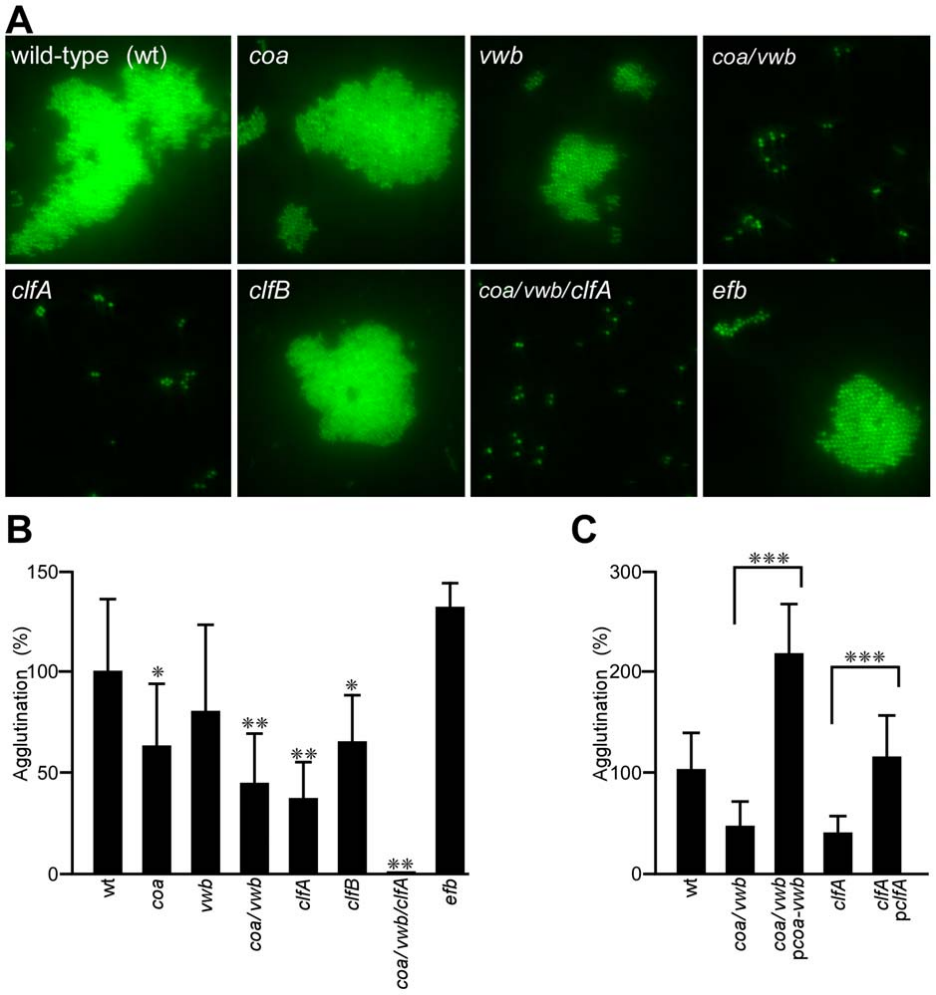
*Staphylococcus aureus* agglutination in citrate-plasma is a multi-factorial process and essential for the pathogenesis of sepsis in mice. (A) Agglutination in EDTA-plasma of Syto-9 stained *S. aureus* Newman wild-type (wt) or its isogenic mutants with insertional lesions in single or multiple genes: *coa* (coagulase), *vwb* (von Willebrand factor binding protein), *clfA* (clumping factor A), *clfB* and *efb* (extracellular fibrinogen binding protein). (B) Quantification of agglutination for staphylococcal mutants (A) expressed as the percent relative to wt (100%). Average and standard error of the means were calculated from sixteen fields of microscopic view and statistical significance was assessed in pairwise comparison between wt and mutant with the two-tailed Student's t-test: *P<0.01, **P<0.0001. (C) Complementation studies of staphylococcal agglutination using the slide agglutination test. *S. aureus* Newman variants *coa*/*vwb* and *clfA* were transformed with plasmids p*coa-vwb* and p*clfA*, respectively. Statistical significance was analyzed by two-tailed Student's *t*-test; ***P<0.0001.

*S. aureus* Newman mutants with defined genetic lesions [29] were screened for defects in agglutination (Fig. 1A). Mutations that abrogated the secretion of only one of the two coagulases, Coa [30] or vWbp [31], had little or no effect on agglutination (Fig. 1AB). In contrast, a mutant lacking both genes (*coa*/*vwb*) was severely impaired for agglutination, similar to a *clfA* variant (Fig. 1AB). A mutant lacking all three genes - *coa*, *vwb*, and *clfA -* was unable to agglutinate in plasma (Fig. 1AB). Mutants with insertional lesions in other known fibrinogen binding proteins, *efb* [32], [33] and *clfB* [34], did not cause large defects in agglutination (Fig. 1AB). The phenotypic agglutination defects of *coa*/*vwb* as well as *clfA* mutants could be restored by transformation of staphylococci with p*coa-vwb* and p*clfA*, respectively, plasmids encoding wild-type alleles to the corresponding mutational lesions (Fig. 1C). Thus, unlike ClfA-mediated clumping of staphylococci via binding to fibrinogen [15], *S. aureus* agglutination appears to be a multi-factorial process involving coagulases, ClfA, as well as fibrinogen and prothrombin [26].

### Staphylococcal agglutination in septic mice

**Figure 2:**
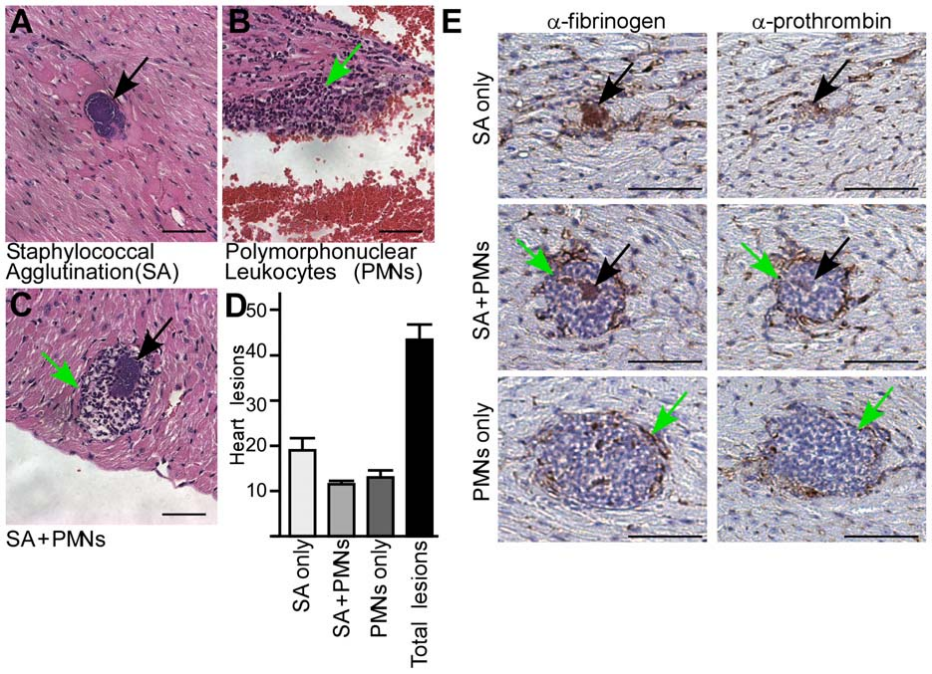
*Staphylococcus aureus* agglutination occurs during the pathogenesis of sepsis in mice. (A–D) Quantification of heart lesions in BALB/c mice (n = 10) 12 hours post-infection with *S. aureus* Newman. Three types of lesions were observed with either (A) staphylococcal agglutination (SA) without immune cell infiltrates (PMNs, polymorphonuclear leukocytes), (B) immune cell infiltrates without SAs (PMNs only) or (C) SA with surrounding granulocytes (SA+PMNs). Heart tissues were stained with hematoxylin-eosin and lesions enumerated (D). Error bars represent standard error of the mean of tissue samples. Data are representative of two independent experiments. (E) Immuno-histochemical analysis of heart tissues from BALB/c mice (n = 10) 12 hours following intravenous challenge with *S. aureus* Newman. Samples were stained with antibodies directed against mouse fibrinogen (α-fibrinogen) or mouse prothrombin (α-prothrombin). Arrows point to staphylococcal agglutinations (black) or immune cell infiltrates (green); scale bars represent 1 µm.

**Figure 3:**
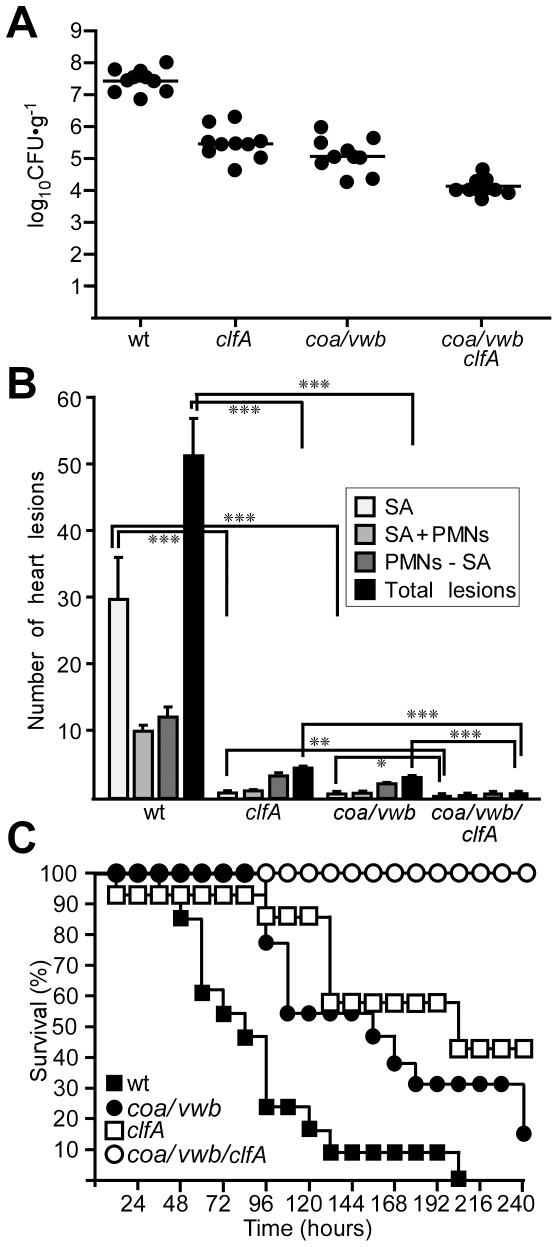
Staphylococcal agglutination in heart tissues is required for the pathogenesis of sepsis. (A) Staphylococcal load, enumerated as colony forming units (CFU), in heart tissues of BALB/c mice (n = 10) 12 hours after retro-orbital inoculation with 10^8^ CFU of *S. aureus* Newman (wt) or its variant strains (*clfA*, *coa*/*vwb* and *coa*/*vwb*/*clfA*). Horizontal lines represent mean CFU. Statistical analysis was performed with the Mann-Whitney test: wt vs. *clfA*, P = 0.0002; wt vs. *coa*/*vwb*, P = 0.0002; wt vs. *coa*/*vwb*/*clfA*, P = 0.0002; *coa*/*vwb* vs. *coa*/*vwb*/*clfA*, P = 0.0007; *clfA* vs. *coa*/*vwb*/*clfA*, P = 0.0002. Data are representative of two independent experiments. (B) Summary of histopathology findings in thin-sectioned and hematoxylin-eosin stained heart tissue from BALB/c mice (n = 10) 12 hours after retro-orbital injection of *S. aureus* Newman wild-type (wt) or its *clfA*, *coa*/*vwb* as well as *clfA*/*coa*/*vwb* variants. Representative lesions in heart tissues included staphylococcal agglutination without PMNs (SA), with PMNs (SA+PMNs), and PMN accumulation without staphylococcal agglutination (PMNs–SA). Error bars represent standard error of the mean from 10 hearts. Statistical significance of lesions for each mutant compared to wt infection was determined by Student's t test: *P<0.05, **P<0.01, ***P<0.001. Data are representative of two independent experiments. (C) Survival of cohorts of BALB/c mice (n = 20) following intravenous injection with *S. aureus* Newman (wt) or variants lacking *coa*, *vwb* or *clfA*. Data are representative of three independent experiments. Statistical significance was assessed with the logrank test: wt vs. *coa*/*vwb* (P<0.01), wt vs. *clfA* (P<0.001), and wt vs. *coa*/*vwb*/*clfA* (P<0.0001).

To test whether staphylococcal agglutination occurred in mice with sepsis, the hearts of animals that had succumbed to *S. aureus* Newman challenge were examined for histopathology (Fig. 2). Deposits of large numbers of staphylococci, mostly without immune cell infiltrates, were identified in hematoxylin-eosin stained heart tissue twelve hours after infection (Fig. 2A–D). The appearance of these staphylococcal agglutinations is consistent with the general concept of thromboembolic deposition of *S. aureus* during sepsis [35] (Fig. 2A). Immuno-histochemical staining was used to detect specific agglutination factors (Fig. 2E). These experiments identified prothrombin and fibrinogen (fibrin) in the immediate vicinity of staphylococcal agglutinations (Fig. 2E). In agreement with the hypothesis that agglutination contributes to the pathogenesis of sepsis, fewer heart lesions were observed when mice were challenged with either *clfA* or *coa*/*vwb* variants (Fig. 3). Of note, heart tissues of animals necropsied twelve hours after intravenous challenge harbored considerable loads of staphylococci, irrespective of the challenge strain. Nevertheless, histopathology features of heart lesions associated with *clfA* or *coa*/*vwb* variants revealed immune cell infiltrates in the absence of staphylococcal agglutinations (Fig. 3B). A mutant lacking all three agglutination factors - *clfA*, *coa* and *vwb* - failed to generate either immune cell infiltrates or *S. aureus* agglutinations in heart tissues (Fig. 3B) and appeared avirulent in the mouse sepsis model (Fig. 3C).

### Clumping factor A tethers staphylococci to fibrin cables

**Figure 4:**
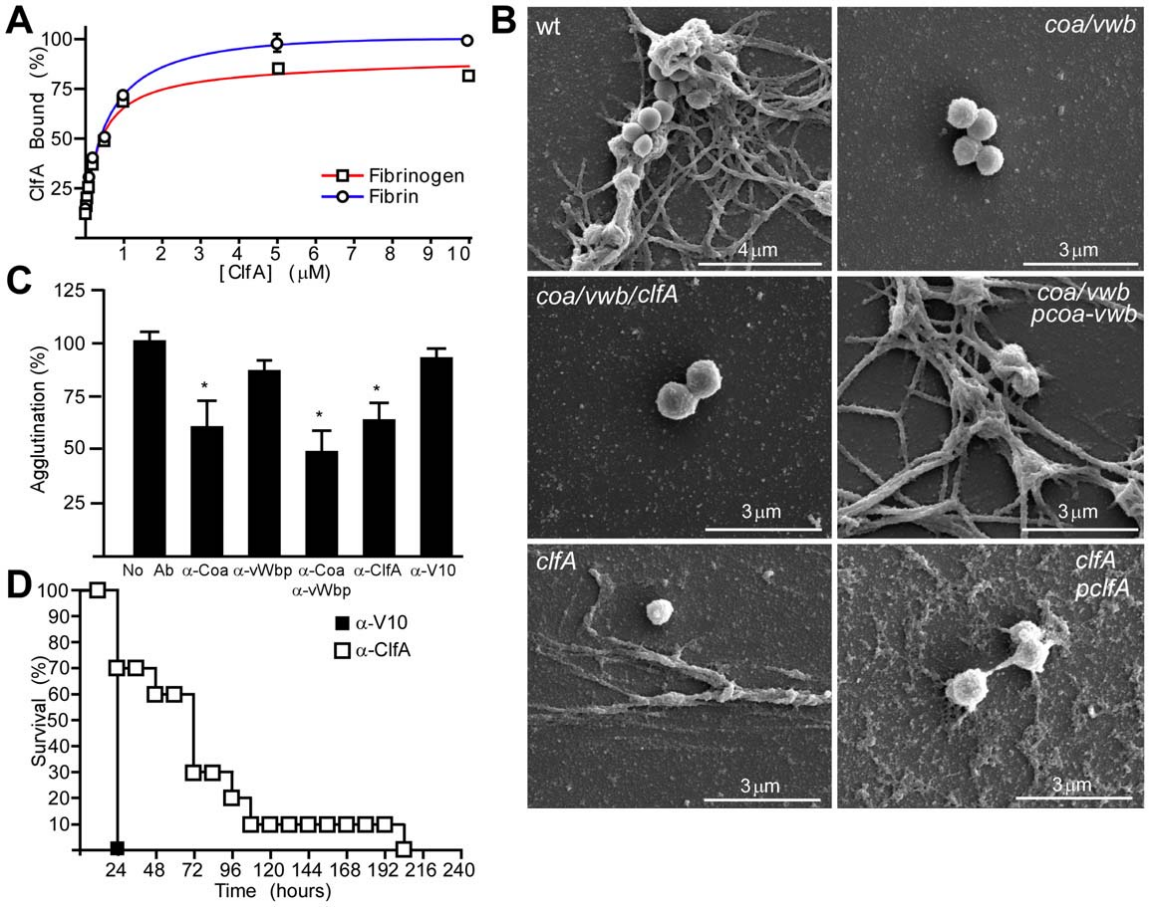
ClfA enables staphylococcal agglutination with fibrin cables *in vitro* and *in vivo*. (A) The association of purified recombinant ClfA with immobilized fibrinogen or fibrin was assessed by ELISA and analyzed as the percentage of maximal binding. Average and standard error of the means were calculated from three independent experiments. Curves represent nonlinear regression for one-site binding saturation performed with GraphPad Prism, Fbgn R^2^ = 0.9876; Fibrin R^2^ = 0.9876. (B) Scanning electron micrographs of *S. aureus* Newman (wt) and its isogenic mutants immersed in plasma. (C) Affinity-purified rabbit IgG specific for Coa (α-Coa), vwb (α-vWb), ClfA (α-ClfA) or the plague protective antigen V10 (α-V10) was analyzed for its ability to prevent staphylococcal agglutination. Statistical significance of antibody effects compared to a mock treated control was assessed with the Student's t test: *P<0.05. (D) BALB/c mice (n = 10) were passively immunized by intraperitoneal injection with affinity-purified antibodies against V10 or ClfA and disease protection assessed by intravenous challenge with *S. aureus* Newman. Data represent one of three independent experiments. Statistical significance was assessed with the logrank test: P<0.01.

Staphylococcal agglutination requires coagulase catalyzed conversion of fibrinogen to fibrin as well as ClfA-mediated attachments. If so, ClfA may bind not only fibrinogen but also fibrin. This prediction was tested by measuring the binding of purified recombinant ClfA to either fibrinogen or fibrin immobilized in wells of polystyrene plates (Fig. 4A). Using non-linear regression analyses, we calculated a dissociation constant (K_d_) of 395.2 nM (±51.82) for ClfA binding to fibrinogen, comparable to earlier affinity measurements [18]. The K_d_ of ClfA binding to fibrin was calculated as 661.9 nM (±80.32), which is not significantly different from the affinity of ClfA for fibrinogen (Fig. 4A). To further investigate *S. aureus* Newman interactions with fibrin, staphylococci were examined by scanning electron microscopy (SEM), which revealed agglutinated wild-type bacteria enmeshed in fibrin cables (Fig. 4B). SEM analysis of the staphylococcal variants *coa*/*vwb* and *coa*/*vwb*/*clfA* identified bacteria without fibrin cables (Fig. 4B). The *clfA* mutant continued to convert fibrinogen to fibrin, however *clfA* variant staphylococci did not agglutinate with fibrin cables (Fig. 4B). Plasmids p*coa-vwb* and p*clfA* complemented the phenotypes caused by mutations in the corresponding genes and restored staphylococcal agglutination to wild-type levels (Fig. 4B). These data are in agreement with our general hypothesis that Coa/vWbp-derived fibrin cables provide a tether for ClfA-mediated staphylococcal agglutination (Fig. 4B).

### Antibodies that prevent staphylococcal agglutination and sepsis

**Figure 5:**
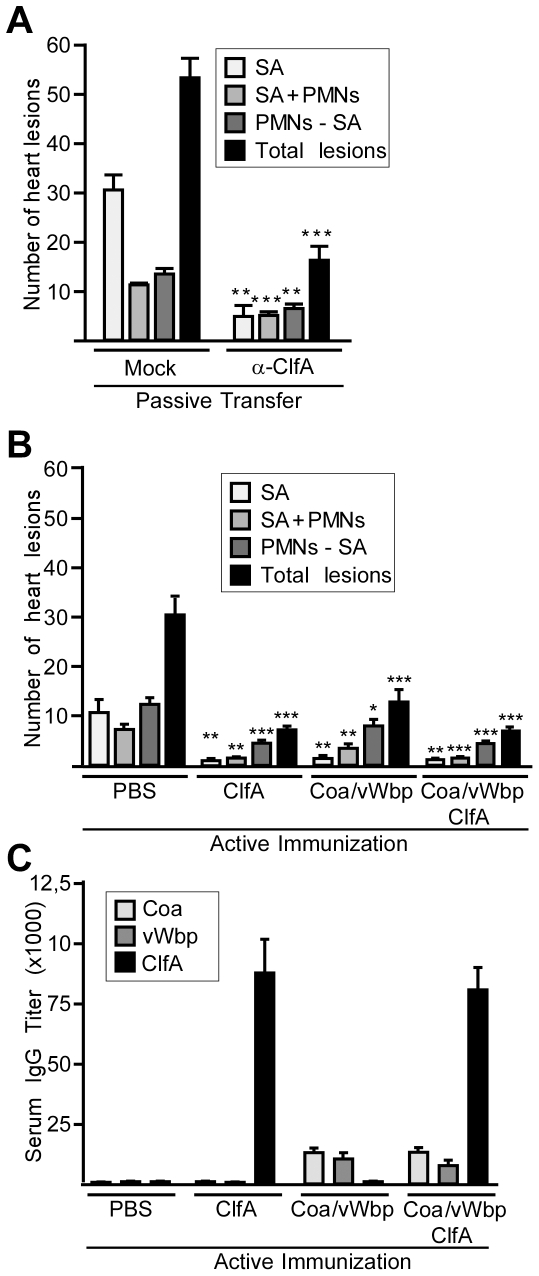
Neutralization of coagulases and ClfA prevents staphylococcal agglutination in heart tissues of septic mice. (A) Quantification of histopathology lesions in heart tissues of BALB/c mice (n = 10) passively immunized with affinity-purified V10 control antibodies (which neutralize the plague protective antigen LcrV) or ClfA antibodies prior to lethal infection. Hearts were removed during necropsy 12 hours after retro-orbital inoculation of staphylococci. Tissues were thin-sectioned, stained with hematoxylin-eosin and histopathology lesions enumerated. Error bars represent standard error of the mean from cohorts of ten mice. Statistical analysis was performed by two-tailed Student's *t*-test comparing same lesion types between mock-immunized and vaccinated animals: *P<0.05, **P<0.01, ***P<0.001. (B) Quantification of three types of histopathology lesions in heart tissues from mice actively immunized with recombinant Coa, vWbp, or ClfA. Hearts were removed during necropsy 12 hours after retro-orbital inoculation of staphylococci into BALB/c mice (n = 10). Tissues were thin-sectioned, stained with hematoxylin-eosin and histopathology lesions enumerated. Error bars represent standard error of the mean from cohorts of ten mice. Statistical analysis was performed by Student's two-tailed *t*-test comparing same lesion types between mock-immunized and vaccinated animals: *P<0.05, **P<0.01, ***P<0.001. Data are representative of two independent experiments. (C) Half maximal IgG antibody titer specific for Coa, vWb or ClfA antigens in serum following active vaccination of BALB/c mice (n = 5). Blood samples were drawn at the time of challenge. Error bars represent standard deviation of serum IgG titers. The limit of detection is 100.

To further explore the contributions of Coa, vWbp and ClfA to staphylococcal agglutination, we raised rabbit antibodies against affinity purified recombinant proteins [12], [36]. Affinity purified rabbit antibodies specific for Coa, vWbp or ClfA inhibited *S. aureus* Newman agglutination in plasma (Fig. 4C). Passive transfer of ClfA-specific rabbit antibodies (85 µg purified antigen-specific IgG) into the peritoneal cavity of mice reduced the deposition of *S. aureus* Newman agglutinations in heart tissues of infected animals (Fig. 5A). Active immunization of mice with purified Coa and vWbp or ClfA raised specific IgG antibodies and reduced the frequency of heart lesions in animals challenged for twelve hours with wild-type *S. aureus* Newman (Fig. 5B). In particular, the abundance of staphylococcal agglutinations without immune cell infiltrates was reduced (Fig. 5B). Active immunization of mice with all three antigens – Coa, vWbp and ClfA – eliminated staphylococcal agglutination in heart tissues and caused the largest reduction of all types of pathological lesions (Fig. 5B). Similar to Coa- and vWbp-specific immunoglobulin [12], passive transfer of ClfA-specific rabbit antibodies into the peritoneal cavity of mice increased the survival time in the sepsis model of infection (Fig. 4D). These data corroborate the concept that ClfA-specific antibodies can improve the outcome of *S. aureus* Newman sepsis [24].

### Direct thrombin inhibitors and staphylococcal sepsis

**Figure 6:**
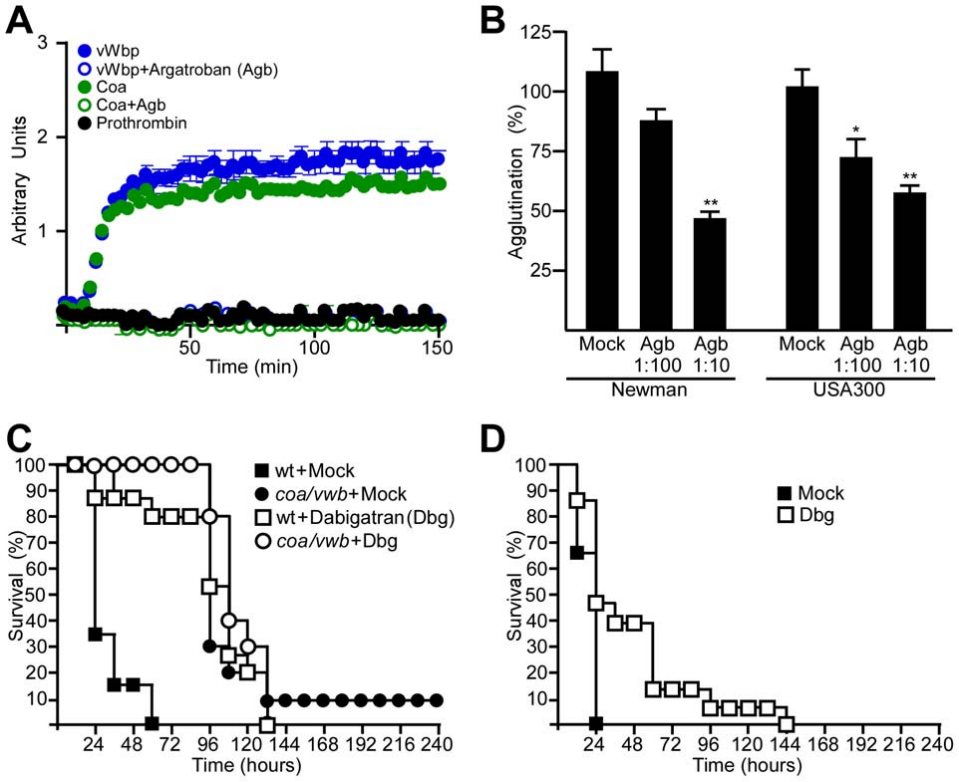
Direct thrombin inhibitors block a key step in staphylococcal pathogenesis. (A) Conversion of fibrinogen to fibrin by prothrombin, Coa·prothrombin or vwb·prothrombin was detected in the presence or absence of 200 ng argatroban (Agb). Arbitrary units are defined as A_450_*100. Average and standard error of the means were calculated from three independent measurements. (B) Agglutination of *S. aureus* Newman or *S. aureus* USA300 LAC in plasma in the presence of increasing concentrations of Agb. Average and standard error of the means were calculated from three independent measurements and statistical significance was assessed with the Student's two-tailed t-test: *P<0.05, **P<0.0001. (C) Survival of cohorts of BALB/c mice (n = 15) treated with saline (mock) or dabigatran-etexilate (Dbg) and infected with either *S. aureus* Newman or the *coa*/*vwb* mutant strain. Statistical significance was analyzed with the logrank test: mock vs. Dbg with wt challenge: P <0.0001; mock vs. Dbg with *coa*/*vwb* challenge: P = 0.43. Data are representative of three independent experiments. (D) Survival of cohorts of BALB/c mice (n = 15) treated with saline (mock) or dabigatran (Dbg) and challenged by intravenous inoculation with *S. aureus* USA300 LAC. Statistical significance was analyzed with the logrank test: mock vs. Dbg, P<0.01. Data are representative of three independent experiments.

Univalent direct thrombin inhibitors, e.g. argatroban and dabigatran, inhibit the proteolytically active Coa·prothrombin complex [37], [38]. We examined whether these inhibitors also block the catalytic activity of vWbp·prothrombin. As a control, conversion of fibrinogen to fibrin by thrombin was monitored as an increase in sample absorbance at 450 nm. Compared to a mock control, this reaction was blocked with 200 ng argatroban (Fig. 6A). Treatment of fibrinogen with either Coa·prothrombin or vWbp·prothrombin led to fibrin conversion, whereas incubation with prothrombin alone did not (Fig. 6A). Incubation of both Coa·prothrombin or vWbp·prothrombin with 200 ng argatroban blocked the conversion of fibrinogen to fibrin (Fig. 6A). Argatroban treatment also interfered with the agglutination of *S. aureus* Newman in plasma (Fig. 6B).

To evaluate the efficacy of direct thrombin inhibitors on the outcome of *S. aureus* Newman sepsis, mice received intraperitoneal injections with 10 mg/kg dabigatran-etexilate in 12 hour intervals. Dabigatran-etexilate is converted in mammalian tissues to its active form, dabigatran, which acts as a direct inhibitor of thrombin [39]. To assess dabigatran activity, mouse blood samples were drawn by cardiac puncture and the dilute thrombin time was determined (Fig. S1). Following challenge of mice via blood stream injection of 1×10^8^ CFU *S. aureus* Newman, mock treated animals died of sepsis within 60 hours post challenge (Fig. 6C). In contrast, dabigatran-etexilate treated animals survived up to 132 hours, albeit that all animals in this cohort eventually succumbed to the challenge (Fig. 6C). To determine whether direct thrombin inhibitors specifically block Coa and vWbp, mock or dabigatran-etexilate treated animals were challenged with the *S. aureus coa*/*vwb* mutant. In these experiments, dabigatran-etexilate treatment had no effect on survival or time-to-death (Fig. 6C). Mock or dabigatran-etexilate treated mice were also infected with lethal doses of *S. aureus* USA300 LAC, the current clone responsible for the epidemic of community-acquired MRSA infections in the United States [5]. Dabigatran-etexilate treatment prolonged the survival of septic mice (Fig. 6D).

### Inhibiting multiple staphylococcal factors improves the outcome of sepsis

**Figure 7:**
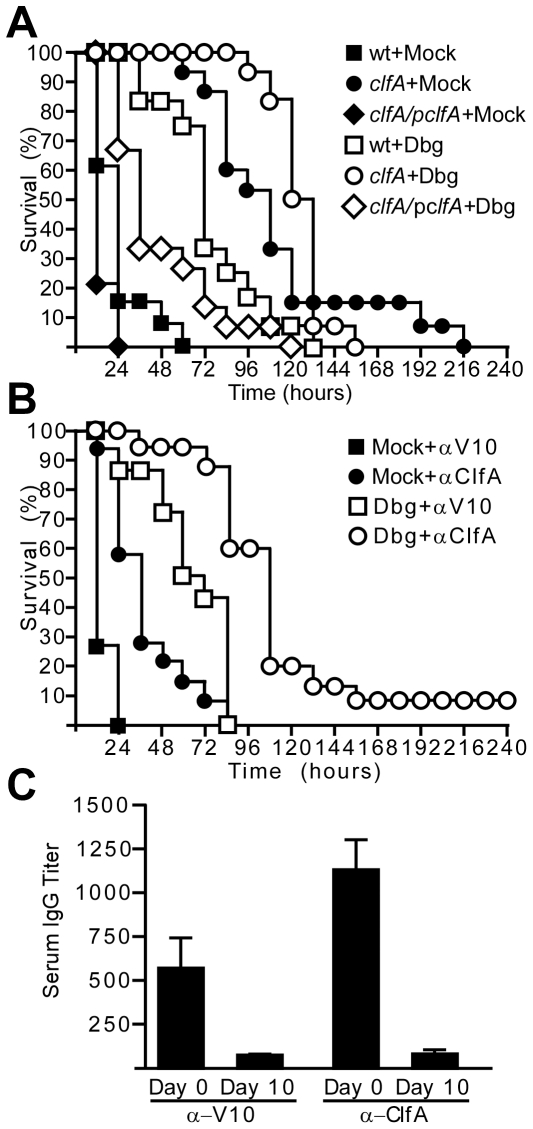
Additive protective effects of direct thrombin inhibitors and ClfA-specific antibodies against *S. aureus* sepsis. (A) Survival of cohorts of BALB/c mice (n = 15) treated with saline (mock) or dabigatran (Dbg) followed by intravenous inoculation with *S. aureus* Newman (wt), *clfA* or *clfA* (pClfA) variants. Data are representative of three independent experiments. (B) Survival of cohorts of BALB/c mice (n = 15) treated with saline (mock) or Dbg and passively immunized (5 mg·kg^−1^) with affinity-purified antibodies against V10 or ClfA. Animals were challenged by intravenous inoculation with *S. aureus* Newman. Statistical analysis was assessed with the logrank test: mock-V10 vs. mock-ClfA, P<0.001; Dbg-mock vs. Dbg-ClfA, P< 0.001. Data are representative of three independent experiments. (C) Half-maximal IgG titer of α-V10 or α-ClfA in serum of passively immunized mice (n = 5) was determined by ELISA. Blood was drawn on day 0, six hours post-immunization and at day 10, when the experiment was terminated.

If *clfA*, *coa* and *vwb* act together to promote *S. aureus* Newman agglutination, dabigatran-etexilate treatment would be expected to improve the outcome of sepsis caused by *clfA* mutant staphylococci (Fig. 7A). Indeed, dabigatran-etexilate treatment increased the survival and time-to-death of mice with sepsis caused by *clfA* mutant *S. aureus* compared to a control strain harboring the complementing plasmid p*clfA* (Fig. 7A). Dabigatran-etexilate treatment further improved the disease outcome of animals challenged with *clfA* mutant staphylococci compared to a cohort of mock treated mice (Fig. 7A). Injection of *clfA* mutants carrying p*clfA* into the blood stream of mice resulted in reduced time-to-death compared to the wild-type parent, *S. aureus* Newman (Fig. 7A). Nevertheless, animals infected with the *clfA* (p*clfA*) variant also benefited from dabigatran-etexilate treatment (Fig. 7A).

To test whether combining dabigatran-etexilate and ClfA-specific antibodies can improve the outcome of staphylococcal sepsis, animals received both treatments followed by challenge with a lethal dose of *S. aureus* (Fig. 7B). As compared to mock-treated animals or mice receiving either dabigatran or ClfA-specific antibodies, the combination of dabigatran and ClfA-specific antibodies led to increased time-to-death and survival of staphylococcal sepsis (Fig. 7BC).

**Figure 8:**
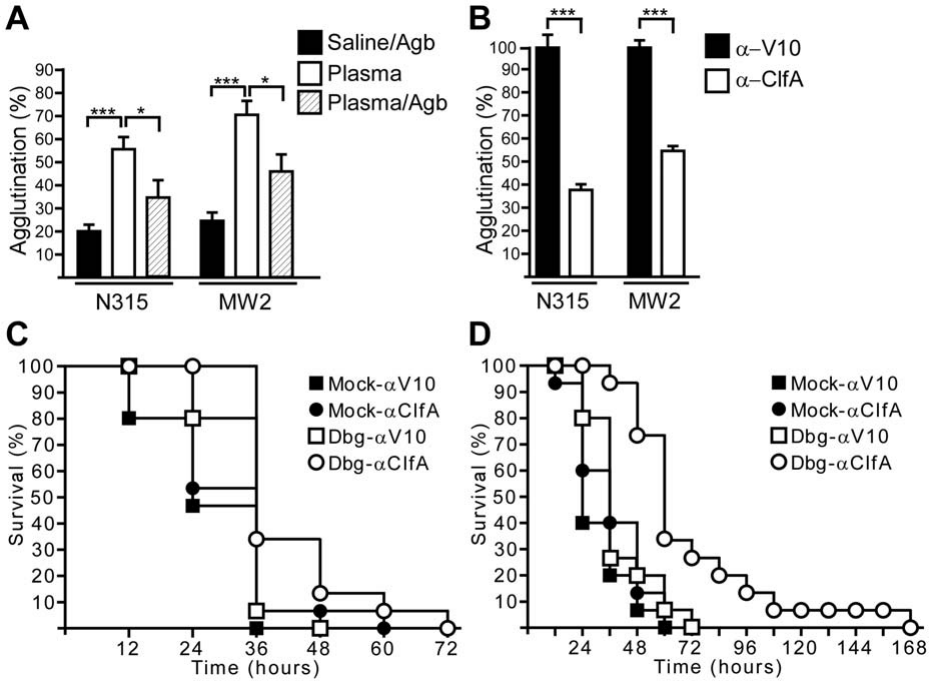
Direct thrombin inhibitors and ClfA-specific antibodies increase the time-to-death of MRSA sepsis in mice. (A) Agglutination of methicillin-resistant *S. aureus* isolates N315 or MW2 in plasma in the presence or absence of argatroban (Agb). Average and standard error of the means were calculated from at least five independent measurements and statistical significance was assessed with the Student's two-tailed t-test: *P<0.05, ***P<0.0001. (B) Affinity-purified rabbit IgG specific for ClfA (α-ClfA) or the plague protective antigen V10 (α-V10) was analyzed for its ability to prevent agglutination of MRSA strains N315 and MW2 in plasma. Average and standard error of the mean were calculated from 16 fields of view from two independent experiments. Statistical significance of antibody effects compared to a mock treated control was assessed with the Student's two-tailed t test: ***P<0.001. (C) Survival of cohorts of BALB/c mice (n = 15) treated with saline (mock) or Dbg and passively immunized (5 mg·kg^−1^) with affinity-purified antibodies against V10 or ClfA. Animals were challenged by intravenous inoculation with MRSA strain N315. Statistical analysis was assessed with the logrank test: mock-V10 vs. mock-ClfA, not significant; mock-V10 vs. Dbg-ClfA, P<0.05; mock-ClfA vs. Dbg-ClfA, P<0.05; Dbg-mock vs. Dbg-ClfA, P<0.01. Data are representative of two independent experiments. (D) Survival of cohorts of BALB/c mice (n = 15) treated as described in panel (C) and challenged by intravenous inoculation with MRSA strain MW2. Statistical analysis was assessed with the logrank test: mock-V10 vs. mock-ClfA, not significant; mock-ClfA vs. Dbg-ClfA, P<0.001; Dbg-mock vs. Dbg-ClfA, P<0.001. Data are representative of two independent experiments.

We wondered whether the use of thrombin inhibitors and ClfA-specific antibodies could aid in the prevention of sepsis caused by clinical *S. aureus* isolates. To test this, we used the community-acquired MRSA isolate MW2, which was isolated from a fatal case of septicemia [40], as well as the hospital-acquired MRSA isolate N315 [41]. *S. aureus* strains N315 and MW2 both agglutinated when suspended in EDTA-plasma (Fig. 8A). These reactions were inhibited by treatment with argatroban (Fig. 8A) or with ClfA-specific antibodies (Fig. 8B). Treatment of mice with both dabigatran and ClfA-specific antibodies led to increased time-to-death during sepsis caused by either *S. aureus* N315 or *S. aureus* MW2 (Fig. 8CD). In contrast, the use of either dabigatran or ClfA-specific antibodies alone did not prolong the survival of mice receiving a lethal challenge of *S. aureus* N315 or *S. aureus* MW2 (Fig. 8CD).

## Discussion

Sepsis is a clinical condition in response to severe bacterial infection, which is associated with high mortality due to continued activation and apoptosis of immune cells and malperfusion of organ systems [42]. During sepsis, the physiological coordination between hemostasis and inflammation is dysregulated, triggering intravascular fibrin deposits [disseminated intravascular coagulation (DIC)] [43]. Dysregulated clotting places sepsis patients at high risk for systemic bleeding and loss of perfusion for vital organ systems [42]. Most anticoagulants, including the thrombin inhibitors heparin, hirudin and antithrombin, cannot alter the outcome of sepsis, likely because these compounds can halt an advancing coagulopathy but are unable to reverse the detrimental effects of established disease [43], [44], [45]. Activated protein C is a serine protease that inactivates the clotting cascade factors Va and VIIIa, without which thrombin-mediated coagulation, i.e. the conversion of fibrinogen to fibrin, is slowed by 2–3 orders of magnitude [46]. Recombinant human activated protein C (drotrecogin alfa) is currently the only FDA-licensed adjunctive therapy in patients with severe sepsis [47], [48]. Clinical trial data indicate reduced mortality in 6.1% of all cases but also considerable bleeding risks. Activated protein C may be particularly useful in preventing sepsis caused by *Escherichia coli* or other Gram-negative bacteria [49]. The ability of activated protein C treatment to affect the outcome of sepsis caused by different bacterial or fungal pathogens is not known [50].

Invasive *S. aureus* infections are frequently associated with bacteremia and may rapidly advance to sepsis [51]. The use of β-lactam antibiotics is obsolete for the treatment of sepsis with drug-resistant *S. aureus* strains (MRSA)[51]. Patients with MRSA infections typically receive vancomycin [51], a glycopeptide antibiotic that blocks bacterial cell wall synthesis [52]. Due to significant nephrotoxicity, vancomycin therapy must be carefully monitored to exceed the minimal inhibitory concentration for staphylococci in host tissues yet avoid the detrimental effects of this compound on kidney function [53]. Even with intensive clinical care, the annual survival of patients with MRSA sepsis is low (<50%) [54]. Thus, preventive measures or therapeutics that improve the outcome of MRSA sepsis represent a pressing public health issue in the United States [51].

*S. aureus* sepsis isolates coagulate blood and/or agglutinate in citrate-plasma [2]. Genome sequencing revealed that all clinical *S. aureus* isolates harbor functional *coa*, *vwb* and *clfA* genes [55]. In contrast, only some staphylococcal strains, those carrying *hlb*-converting phages, harbor the *sak* gene [56], whose secreted product staphylokinase associates with plasminogen to promote fibrinolysis [57], [58] in addition to cleaving human antimicrobial peptides (defensins)[59]. We wondered whether the unique attributes of *S. aureus* to generate fibrin deposits contribute also to the pathogenesis of sepsis. Using a mouse model for this disease, we observed that sortase A mutants, unable to anchor any one of nineteen different surface proteins in the staphylococcal envelope [60], were unable to cause sepsis [27]. ClfA, a surface protein associated with bacterial binding to fibrinogen [17], is the most important sortase A anchored virulence factor for sepsis (Table 1). Mutations in two coagulases, *coa* and *vwb* [12], further diminish the virulence of *clfA* mutants to a level that resembles that of sortase A variants (Fig. 1). To investigate the physiological role of coagulases and ClfA, we studied staphylococcal agglutination, a clinical microbiological assay requiring fibrinogen and prothrombin for bacterial association with fibrin fragments [26]. All three staphylococcal products - Coa, vWbp and ClfA – were required to agglutinate the pathogen in blood and cause lethal disease in mice. We presume that staphylococcal agglutination *in vivo* promotes the formation of thromboembolic lesions that contribute to the rapid lethality of *S. aureus* Newman [61] or USA300 LAC infections [62] into the bloodstream of mice.

Pretreatment of animals with dabigatran-etexilate, which blocks cleavage of fibrinogen by Coa·prothrombin and vWbp·prothrombin, as well as administration of ClfA-specific antibodies both interfere with *S. aureus* agglutination and reduce the mortality of sepsis. Nevertheless, combining direct thrombin inhibitors with ClfA-specific antibodies or combining anti-ClfA with anti-Coa/anti-vWbp can generate an even higher level of protection against sepsis. The recent licensure of direct thrombin inhibitors (e.g. dabigatran) and the availability of ClfA-specific monoclonal antibody provide an opportunity for the rapid testing of such regimen to reduce the incidence and/or the mortality of *S. aureus* sepsis.

ClfA binding to the C-terminal residues of the fibrinogen γ-chain may not be involved in *S. aureus* agglutination; this portion of the polypeptide is thought to be buried within polymerized fibrin cables [63], [64]. If so, staphylocoagulase mediated cleavage of fibrinogen may reveal another binding site for ClfA on the surface of fibrin cables, enabling staphylococcal agglutination in a manner that can be inhibited with ClfA-specific antibodies. Direct thrombin inhibitors block coagulase (Coa and vWbp) mediated cleavage of fibrinogen and thereby hinder the formation of ClfA binding sites on the surface of fibrin cables. These compounds also mimic the phenotype of *S. aureus* Newman coagulase mutants (Δ*coa*, *vwb*) in the murine sepsis model and presumably exert a similar effect in preventing the formation of staphylococcal abscess.

*S. aureus* is a frequent cause of human wound infections [1], however the contribution of coagulases towards the establishment of this disease is not known. Of note, physiological hemostasis during wound healing generates fibrin and platelet deposits within wounded tissues [65]. Thus, it would be interesting to explore whether preventive treatment with direct thrombin inhibitors as well as ClfA-specific antibodies can reduce the incidence of hospital-acquired sepsis and/or wound infections.

## Materials and Methods

### Ethics statement

Animal experiments involving *S. aureus* challenge followed protocols that were reviewed, approved and performed under the regulatory supervision of The University of Chicago's Institutional Biosafety Committee (IBC) and the Institutional Animal Care and Use Committee (IACUC). Animals were managed by the University of Chicago Animal Resource Center, which is accredited by the American Association for Accreditation of Laboratory Animal Care and the Department of Health and Human Services (DHHS number A3523-01). Animals were maintained in accordance with the applicable portions of the Animal Welfare Act and the DHHS “Guide for the Care and Use of Laboratory Animals”. Veterinary Care was under the direction of full-time resident veterinarians boarded by the American College of Laboratory Animal Medicine. BALB/c mice and New Zealand white rabbits were purchased from Charles River Laboratories and Harlan Sprague Dawley, respectively. After confirming that the data sets abide by a normal distribution, the statistical analysis of staphylococcal sepsis was analyzed using the two-tailed Logrank test. Quantification of mouse heart tissue histopathology was analyzed for statistical significance using the unpaired two-tailed Student's t-test. The bacterial load (CFU) in heart tissue from mice infected with staphylococcal variants was analyzed with the Mann Whitney test. The results of all animal experiments were examined for reproducibility.

### Bacterial strains and growth of cultures

*S. aureus* strains Newman [61], USA300 LAC [62], MW2 [40] and N315 [41] were cultured on tryptic soy agar or broth at 37°C. *E. coli* strains DH5α and BL21 (DE3) were cultured on Luria Bertani agar or broth at 37°C. Ampicillin (100 µg/ml) and chloramphenicol (10 µg/ml) were used for pET15b and pOS1 selection [66], respectively.

### Transposon mutants and plasmids

Insertional mutations carrying the *bursa aurealis* transposon with an erthyromycin resistance cassette from the *Phoenix* library [29] were transduced with bacteriophage into *S. aureus* Newman or the *coa*/*vwb* mutant [12]. Mutations were verified by PCR with specific primer pairs for *coa* (CGCGGATCCATAGTAACAAAGGATTATAGTGGGAAATCACAAG and TCCCCCGGGTTATTTTGTTACTCTAGGCCCATATGTCGC), *vwb* (CGCGGATCCGTGGTTTCTGGGGAGAAGAATCC and TCCCCCGGGTTTGCAGCCATGCATTAATTATTTGCC) and *clfA* (CGCGGATCC-AAGGTCAAATCGACCGTT and CGGGGTACC-TTATTTCTTATCTTTATTTTCTTTTTTTC) as well as by immunoblotting with specific rabbit antibodies [12], [36]. Complementing plasmids p*coa-vWbp* and p*clfA* were described previously [12], [67]. For immunoblot analysis, 1 mL of staphylococcal overnight cultures grown in tryptic soy broth (Difco) were centrifuged at 8,000×*g* for 3 min in a table top centrifuge and the supernatant was recovered. Proteins in culture supernatants were precipitated with 10% trichloroacetic acid on ice for 20 minutes. Pellets were washed once in 1 mL TSM (100 mM Tris-HCl, pH 7.5, 0.5 M sucrose, 10 mM MgCl_2_), suspended in 500 µL TSM, incubated with 50 µg lysostaphin for 15 minutes at 37°C for 15 minutes. 10% TCA was added and samples were incubated on ice for 10 min. All samples were centrifuged and washed with 1 mL ice-cold 100% acetone. Samples were air dried and solubilized in 75 µL sample buffer (4% SDS, 50 mM Tris-HCl, pH 8.0, 10% glycerol, and bromophenol blue).

### Scanning electron microscopy

Staphylococcal strains were grown to mid-log phase (OD_600_ 0.5), washed twice and suspended in PBS to a final OD_600_ 1. Bacteria were mixed with EDTA-chelated rabbit plasma (1:1) and incubated for 15 minutes. Samples were fixed for 60 minutes in 2% glutaraldehyde in phosphate buffered saline (PBS) at room temperature onto freshly prepared poly-L-lysine coated glass coverslips. Samples were washed twice with PBS and subsequently serially dehydrated by consecutive incubations in 25% and 50% ethanol/PBS, 75% and 90% ethanol/H_2_O, 2× 100% ethanol, followed by 50% ethanol/hexamethyldisilazane (HDMS) and finally with 100% HDMS. After overnight evaporation of HDMS at room temperature, samples were mounted onto specimen mounts (Ted Pella, Inc.) and coated with 80% Pt/20% Pd to 8 nm using a Cressington 208HR Sputter Coater at 20mA prior to examination with a Fei Nova NanoSEM 200 scanning electron microscope. The SEM was operated with an acceleration voltage of 5 kV and samples were viewed at a distance of 5 mm.

### Protein purification

*E. coli* BL21(DE3) harboring expression vectors containing *coa*, *vwb*, or *clfA* were grown at 37°C and induced with 1 mM IPTG after two hours. Three hours following induction, cells were centrifuged at 7,000×*g*, suspended in column buffer (0.1 M Tris-HCl, pH 7.5, 0.5 M NaCl) and lysed in a French pressure cell at 14,000 lb/in^2^. Lysates were subjected to ultracentrifugation at 40,000 ×*g* for 30 min and the supernatant was subjected to Ni-NTA chromatography, washed with column buffer containing 10 mM imidazole, followed by elution with 500 mM imidazole. Eluates were dialyzed against PBS. To remove endotoxin, 1∶100 Triton-X114 was added and the solution was chilled for 10 min, incubated at 37°C for 10 min, and centrifuged at 13,000 ×*g*. This was repeated twice. Supernatant was loaded onto a HiTrap desalting column to remove remnants of Triton-X114. Purity was verified by SDS-PAGE analysis and Coomassie Brilliant Blue staining.

### Rabbit antibodies

Protein concentration was determined using a BCA kit (Pierce). Purity was verified by SDS-PAGE analysis and Coomassie Brilliant Blue staining. Six month old New-Zealand white female rabbits were immunized with 500 µg protein emulsified in CFA (Difco) for initial immunization or IFA for booster immunizations on day 24 and 48. On day 60, rabbits were bled and serum recovered for immunoblotting or passive transfer experiments. For antibody purification, recombinant His_6_-Coa [12], His_6_-vWbp [12], or His_6_-ClfA (5 mg) [36] was covalently linked to HiTrap NHS-activated HP columns (GE Healthcare). This antigen-matrix was then used for affinity chromatography of 10–20 ml of rabbit serum raised against Coa [12], vWbp [12] or ClfA at 4°C. Charged matrix was washed with 50 column volumes of PBS, antibodies eluted with elution buffer (1 M glycine pH 2.5, 0.5 M NaCl) and immediately neutralized with 1 M Tris-HCl, pH 8.5. Purified antibodies were dialyzed overnight against PBS, 0.5 M NaCl at 4°C.

### Agglutination assay

Overnight cultures of staphylococcal strains were washed in 1 mL 0.85% NaCl and suspended to a final concentration of OD_600_ 4.0 in 1 mL. Bacteria were incubated with 1∶500 Syto9 (Invitrogen) for 15 minutes, washed with 1 mL 0.85% NaCl, and suspended in 1 mL saline. Bacteria were mixed 1∶1 with EDTA-chelated rabbit plasma (Becton, Dickinson) on a glass microscope slide and incubated for 15 minutes. Samples were viewed and images captured on an Olympus Provis microscope using a 40× objective. For quantification of agglutination, plasma and bacteria were inoculated onto polystyrene C-Chip disposable hemocytometer slides (IN-CYTO). Brightfield images from sixteen fields of view were taken of bacterial strains using a Nikon TE2000 U with a 20× objective. To determine the degree of agglutination, the Threshold Function in ImageJ software was used to convert the image into a binary image, in which staphylococci are black and the background is white. The mean intensity of the image was measured. The average mean intensity of *S. aureus* Newman in saline without plasma was subtracted from all values and percent agglutination was calculated by normalizing all mean intensity values to *S. aureus* Newman in plasma. To assess the inhibitory affect of antibodies on agglutination, affinity-purified antibodies were incubated with staphylococci to a final concentration of 3 µM for 10 minutes prior to mixture with plasma. To assess the inhibitory affect of argatroban on agglutination, argatroban was diluted 1∶10 and 1∶100 in plasma and incubated for 10 minutes prior to mixture with bacteria. Percent agglutination was measured compared to bacteria in plasma without argatroban. For experiments using *S. aureus* N315 and MW2, agglutination was measured as percent change in OD_550_ following two hours incubation of bacteria with saline containing argatroban (1 mg/mL), plasma, or plasma containing argatroban (1 mg/mL). Error bars represent standard error of the mean from at least three independent experiments to ensure reproducibility.

### Sepsis

Overnight cultures of staphylococcal strains were diluted 1∶100 into fresh TSB and grown until they reached an OD_600_ of 0.4. Bacteria were centrifuged at 7,000 ×*g*, washed, and suspended in the one-tenth volume of PBS. Six week-old female BALB/c mice (n = 15) (Charles River) were injected retro-orbitally with 1×10^8^ CFU (*S. aureus* Newman, MW2, and N315) or 5×10^7^ CFU (*S. aureus* USA300) suspensions in 100 µl of PBS. Mice were monitored for survival over 10 days. To enumerate staphylococcal load in heart tissue twelve hours post-infection, mice were euthanized by CO_2_ asphyxyation and hearts were removed during necropsy. Heart tissue was homogenized in PBS, 0.1% Triton X-100. Serial dilutions of homogenate were spread on TSA and incubated for colony formation. The bacterial load in organ tissue was analyzed in pairwise comparisons between wild-type and mutant strains with the unpaired two-tailed Student's *t-*test. For histopathology, mice infected with *S. aureus* were euthanized 12 hours after infection. Hearts were removed during necropsy and fixed in 10% formalin for 24 hours at room temperature. Tissues were embedded in paraffin, thin-sectioned, stained with hematoxylin and eosin, and examined by light microscopy to enumerate pathological lesions per organ. Data were analyzed in pairwise comparisons between wild-type and mutant strains with the unpaired two-tailed Student's *t*-test. For immunohistochemical analysis, thin-sectioned heart tissues were stained with polyclonal antibodies against mouse prothrombin (Haematologic Technologies) or mouse fibrinogen (Haematologic Technologies).

### ClfA binding to fibrinogen and fibrin

MaxSorb 96-well ELISA plates (Nunc) were coated with human fibrinogen (Sigma) overnight. Wells were washed and solutions of PBS or alpha-thrombin (Innovative Research), 100 nM in 1% sodium-citrate/PBS were added for one hour at room temperature to generate fibrinogen and fibrin wells respectively. As controls, the same conditions were generated in Eppendorf tubes. Following incubation with or without alpha-thrombin, samples were centrifuged at 13,000 ×*g* for 10 min and supernatants were recovered. The sediment was dissolved in 8 M urea. Running buffer (3 M urea, 4% SDS, 10% BME) was added 1:1. Proteins in supernatants and pellets were separated by SDS-PAGE (15%) and stained with Coomassie Brilliant Blue to analyze soluble fibrinogen in the supernatant fraction and fibrin in the sediment. Purified recombinant ClfA in 1% sodium-citrate was added at increasing concentrations to 96-well plates and incubated for one hour. Samples were incubated with polyclonal anti-ClfA (1:1,000) to detect bound-ClfA followed by goat anti-rabbit-HRP (1:10,000). The wells were developed using an OptEIA kit (BD Lifesciences) and absorbance at 450 nm was measured. Non-linear regression assuming one-site saturation kinetics was performed using GraphPad Prism.

### Active immunization

Three week-old BALB/c mice (n = 10) were injected with 50 µg protein emulsified in 100 µl complete Freund's adjuvant. Eleven days post vaccination these mice were boosted with 50 µg protein each emulsified in 100 µl incomplete Freund's adjuvant. On day 21, mice were injected with 1×10^8^ CFU of *S. aureus* challenge strains.

### Passive transfer of antibodies

Six hours prior to infection, six week old BALB/c mice (n = 15) were injected intraperitoneally with specific rabbit antibodies affinity-purified on ClfA- or V10-coupled resin (control IgG specific for the LcrV plague antigen) at a dose of 5 mg/kg body weight. Control mice (n = 5) that received the same antibody via passive transfer were anesthetized and bled retro-orbitally at the time of infection and again at the end of the experiment. Blood was collected using micro-hematocrit capillary tubes (Fisher) in Z-Gel microtubes (Sarstedt). Tubes were centrifuged at 8,000 ×*g* for three minutes, and serum was collected. Antibody titer was measured by ELISA as previously described [27].

### Coagulase activity

Purified recombinant Coa or vWbp (100 nM) were mixed with human prothrombin (Innovative Research) in 1% sodium-citrate/PBS. After an initial reading, fibrinogen (3 µM) (Sigma) was added and conversion of fibrinogen to fibrin was measured as an increase in turbidity at 450 nm in a plate reader (BioTek) at 2.5 min intervals. As controls, the enzymatic activity of human alpha-thrombin (Innovative Research) or prothombin alone were measured. Argatroban (200 ng, Novaplus) was added to reactions prior to the addition of fibrinogen.

### Dabigatran etexilate treatment

Dabigatran (Boehringer Ingelheim) tablets were dissolved in 0.9 N saline and doses of 10 mg/kg in 100 µL were administered. Mice (n = 15) were injected intraperitoneally starting 24 hours prior to infection and continuing every twelve hours during the course of the infection. Control mice received injections of 0.9 N saline. To measure dilute thrombin time, mice (n = 5) received saline or dabigatran treatment and were euthanized by CO_2_ asphyxiation at the time of infection. Blood was drawn by cardiac puncture, diluted in sodium-citrate (1%), centrifuged at 1,500 ×*g* for 5 minutes, and plasma diluted in pooled fresh human plasma 1:6. Thrombin time was measured on a STA-R analyzer (Diagnostica Stago).

## Supporting Information

Figure S1 **Direct thrombin inhibitors and their effect on** ***in vitro*** **and** ***in vivo*** **coagulation.** (A) Conversion of fibrinogen to fibrin by human alpha-thrombin was measured in the presence or absence of 200 ng argatroban. Human prothrombin was incubated alone as negative control. One arbitrary unit is defined as A_450_*100. Error bars represent standard deviation of triplicate experiments. (B) Dilute thrombin time was measured for plasma from mice treated with saline (mock) or Dabigatran-etexilate (Dbg) on the day of infection or on day 10 following infection. Each symbol represents a blood sample from a single mouse. Horizontal lines indicate mean thrombin time for the cohort. Statistical significance was determined by two-tailed Student's *t*-test: *P<0.01, **P<0.001.
(PDF)
