# Supplementary material for: Preventing Staphylococcus aureus Sepsis through the Inhibition of Its Agglutination in Blood
Source: PLoS Pathog. 2011 Oct 20;7(10):e1002307. doi: 10.1371/journal.ppat.1002307 (PMC3197598; doi:10.1371/journal.ppat.1002307)
Supplement: Figure S1 — Direct thrombin inhibitors and their effect on in vitro and in vivo coagulation. (A) Conversion of fibrinogen to fibrin by human alpha-thrombin was measured in the presence or absence of 200 ng argatroban. Human prothrombin was incubated alone as negative control. One arbitrary unit is defined as A450*100. Error bars represent standard deviation of triplicate experiments. (B) Dilute thrombin time was measured for plasma from mice treated with saline (mock) or Dabigatran-etexilate (Dbg) on the day of infection or on day 10 following infection. Each symbol represents a blood sample from a single mouse. Horizontal lines indicate mean thrombin time for the cohort. Statistical significance was determined by two-tailed Student's t-test: *P<0.01, **P<0.001. (PDF) [file ppat.1002307.s001.pdf]

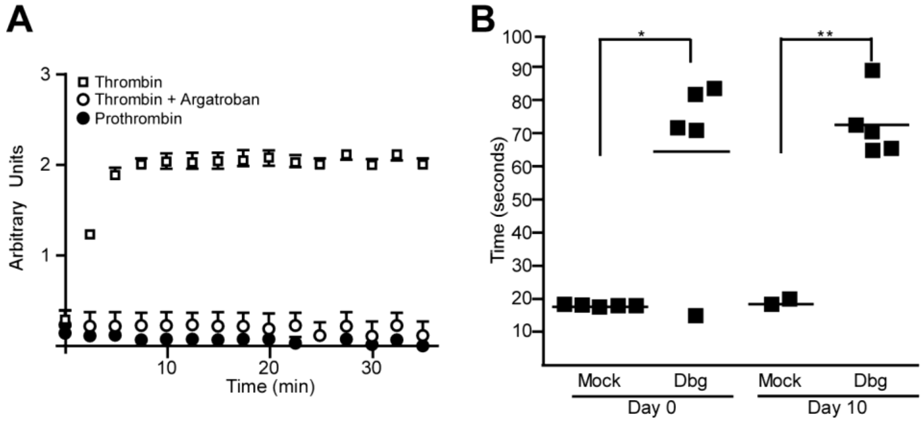

Figure S1. Direct thrombin inhibitors and their effect on *in vitro* and *in vivo* coagulation. (A) Conversion of fibrinogen to fibrin by human alpha-thrombin was measured in the presence or absence of 200 ng argatroban. Human prothrombin was incubated alone as negative control. One arbitrary unit is defined as A_{450}*100. Error bars represent standard deviation of triplicate experiments. (B) Dilute thrombin time was measured for plasma from mice treated with saline (mock) or Dabigatran-etexilate (Dbg) on the day of infection or on day 10 following infection. Each symbol represents a blood sample from a single mouse. Horizontal lines indicate mean thrombin time for the cohort. Statistical significance was determined by two-tailed Student's *t*-test: *P<0.01, **P<0.001.
